# Changes in regional variation in mortality over five decades – The contribution of age and socioeconomic population composition

**DOI:** 10.1016/j.ssmph.2021.100850

**Published:** 2021-06-19

**Authors:** Ulla Suulamo, Lasse Tarkiainen, Hanna Remes, Pekka Martikainen

**Affiliations:** Population Research Unit, Faculty of Social Sciences, University of Helsinki, Unioninkatu 35 (P.O. Box 18), FIN-00014, Helsinki, Finland

**Keywords:** Mortality, Health inequalities, Regional variation, Long-term trends, Multilevel modelling

## Abstract

Existing evidence suggests that within-country area variation in mortality has increased in several high-income countries. Little is known about the role of changes in the population composition of areas in these trends. In this study, we look at mortality variation across Finnish municipalities over five decades. We examine trends by sex, age categories and two broad cause of death groups and assess the role of individual-level compositional factors. Analyses rely on individual-level register data on the total Finnish population aged 30 years and over. We estimated two-level Weibull survival-models with individuals nested in areas for 10 periods between 1972 and 2018 to assess municipal-level variation in mortality. Median hazard ratio (MHR) was used as our summary measure and analyses were adjusted for age and socioeconomic characteristics. The results show a clear overall growth in area variation in mortality with MHR increasing from 1.14 (95% CI 1.12–1.15) to 1.28 (CI 1.26–1.30) among men and 1.17 (CI 1.15–1.18) to 1.30 (CI 1.27–1.32) among women. This growth, however, was fully attenuated by adjustment for age. Area differentials were largest and increased most among men at ages 30–49, and particularly for external causes. This increase was largely due to increasing differentiation in the socioeconomic composition of municipalities. In conclusion, our study shows increases in mortality differentials across municipalities that are mostly attributable to increasing differentiation between municipalities in terms of individual compositional factors.

## Highlights

• Overall, unadjusted municipal-level mortality variation increased over five decades.
• This increase was mainly driven by growing differences in municipal age composition.
• Age-adjusted mortality differentials increased among men aged 30–49 years.
• This increase was fully attenuated after socioeconomic factors were accounted for.
• Municipal variation remained stable and modest at ages 70+ for both men and women.

## 1 Introduction

National-level measures of mortality often hide important within-country differentials. Recent studies provide evidence that while in some countries these differentials have narrowed over time, in others they have persisted and even grown even as overall mortality levels have steadily declined (Vierboom et al., 2019; Pearce & Dorling, 2006; Leyland, 2004; Wilson et al., 2020). Reasons for the developments are, however, unclear. Deindustrialisation constitutes a key feature of social change over the past decades, which has affected areas unevenly leading to growing regional differentiation and changes in the spatial population distribution (Bontje & Musterd, 2012; Martinez-Fernandez & Noya, 2012; Oswalt et al., 2006). We propose that these processes may be associated with the reported trends in within-country mortality variation.

Area differentials in mortality have been documented at different levels ranging from the immediate local area to broader regions, but relatively little evidence exists on long-term trends. In the United States, variation measured at county (Ezzati et al., 2008), state (Wilmoth, Boe, & Barbieri, 2010) and wider geographical (Vierboom et al., 2019) levels has increased from the 1980s. For example, the gap between counties with the lowest and highest life expectancy increased from approximately 10 years–15 years between 1985 and 2010 (Wang et al., 2013). A widening gap between low and high mortality areas in 1980–2000 has also been observed between health districts in New Zealand (Pearce & Dorling, 2006) and regional health authorities in Canada (Mustard et al., 1999). For European countries, the existing evidence points in opposite directions. In Germany (van Raalte et al., 2020), Austria (Gächter & Theurl, 2011) and Norway (Skaftun et al., 2018), differentials have narrowed, whereas widening differentials have been found, for example, in Britain (Leyland, 2004), Spain (Montero-Granados et al., 2007) and Poland (Muszynska et al., 2014). In some countries, variation between areas appears stable, including the Netherlands in 1988–2009 (Janssen et al., 2016) and Sweden and Finland in 1990–2014 (Wilson et al., 2020). Evidence on whether the observed trends differ by age and sex is limited and inconclusive. While larger increases in differentials have been reported among younger men in Germany (van Raalte et al., 2020) and Britain (Smith et al., 2002), important increases among older adults have been reported in France (Bonnet & d’Albis, 2020), Russia (Timonin et al., 2017) and the US (Vierboom & Preston, 2020).

Much of the discussion in the literature interprets area variation in mortality as arising from two principal sources. Compositional effects suggest that differences between areas relate to the uneven spatial distribution of people with different mortality risk due to individual-level characteristics such as age or socioeconomic position, while contextual effects originate from characteristics and processes occurring at the area level (Subramanian et al., 2003). Even though the extent to which these two effects can be separated is arguable (Macintyre et al., 2002), they offer a useful analytical framework for examining how both individual characteristics and contextual circumstances manifest in mortality differences between areas.

Previous studies have rarely attempted to identify reasons for the reported long-term trends in area-level mortality differentials, but some potential explanations and associated factors have been identified; these include changes in the importance of area-level socioeconomic conditions (Kibele et al., 2015) or changing social policies (Gächter & Theurl, 2011; Montero-Granados et al., 2007; Pearce & Dorling, 2006). Furthermore, results from cause-specific analysis suggest that the trends are closely related to changes in the prevalence of health behaviour between areas (Myrskylä & Scholz, 2013; Muszyńska et al., 2014; Timonin et al., 2017; Vierboom et al., 2019).

What we believe is noteworthy is that most prior research has not discussed the contribution of changes in individual-level age- and socioeconomic characteristics of the population, although empirical findings suggest their contribution on mortality variation is often more significant compared to area characteristics (Pickett & Pearl, 2001). In the context of increasing regional differentiation of the past decades in terms of spatial population distribution − most importantly through selective migration of young people from peripheral areas towards urban centres − the role of changes in compositional factors is however particularly relevant. Local population structures directly affect the need and provision of services at local levels. Especially areas with high out-migration face a challenging combination of increasing health care needs of an aging population and a decreasing proportion of younger people to both provide and finance such services as taxpayers. Such discrepancies may affect the availability of adequate services and eventually lead to increasing regional disparities in health and mortality. Therefore, besides from shedding light into the role of changing population composition of areas behind trends in regional mortality variation, research on this topic provides important information for policymakers.

This paper analyses trends in within-country mortality variation in Finland at ages 30 and above in the period 1972–2018. While trends in area level mortality variation have mostly been studied in an ecological setting and with aggregate data, we add to the literature by using individual level register data and a multilevel methodology that integrates the ecological approach with individual risk factor epidemiology (Pickett & Pearl, 2001). Using total population data spanning almost 50 years, we explore long-term trends in municipal-level variation in all-cause mortality among men and women, assess variation in these trends across age categories and internal and external causes of death, and examine the contribution of age and socioeconomic characteristics of the population to the trends.

## 2 Methods

### 2.1 Data

We used individual-level data for the total Finnish population from 1972 to 2018. Socioeconomic and demographic data from censuses and administrative registers were linked by Statistics Finland to death records using personal identification numbers. We included individuals who were 30 years or older at the end of the baseline years 1971, 1975, 1980, 1985, 1990, 1995, 2000, 2005, 2010 and 2015 and followed them for mortality in the periods 1972–75, 1976–80, …, 2011–2015, and 2016–2018. Individuals were followed until death or end of follow-up. Altogether, the final dataset comprised 138,026,531 person-years and 2,236,420 deaths between 1972 and 2018.

We used municipality as the area unit since municipalities are the regional units responsible for the implementation of health services. The number and boundaries of the municipalities have changed from the 1970s. For consistency over time, we used the classification as of 2019 with a total of 311 units. In 1971, the total population of the municipalities varied from 173 to 509,256, with a median of 14,766. In 2015, the corresponding numbers were 99–628,168, and 17,643.

We studied all-cause mortality as well as internal and external mortality based on Statistics Finland's time series cause of death classification which harmonises across different revisions of the International Classification of Diseases (ICD). Death was defined as external if the underlying cause was alcohol-, injury- or violence-related (ICD-10: F10, G312, G4051, G621, G721, I426, K292, K70, K852, K860, O354, P043, Q860, V01–Y89). All other causes were defined as internal.

We adjusted the analyses for the following individual-level variables: age (in five-year groups), education level, occupational social class, and income, all measured at baseline. Education was classified as basic or lower (International Standard Classification of Education ISCED 2011 codes 0–2), secondary (ISCED 3–4) and higher (ISCED 5–8). Occupational class was divided into upper non-manual employees, lower non-manual employees, manual workers, employers/self-employed, and others. Household taxable income was used as our income measure and categorised into quintiles separately for men and women aged 30 and over. To adjust for household size, we divided the household income by the square root of the number of household members (OECD, 2013).

### 2.2 Statistical analysis

We estimated two-level Weibull survival-models, with municipal-level random intercepts and individuals nested within municipalities to assess the contribution of the municipality to the within-country variation. To examine how the variation has evolved over time, we studied mortality in 10 periods between 1972–1975 and 2016–2018. As our main summary measure of municipal-level mortality variation, we used the median hazard ratio (MHR). MHR allows one to quantify the magnitude of the general contextual effects (Austin et al., 2017). The general contextual effect is the correlation between individuals' survival within the same context, in our case municipality (Merlo et al., 2018). This is often measured using the intraclass correlation for continuous outcomes, but MHR is suitable for survival studies (see Austin et al., 2017). MHR indicates the median ratio when comparing the hazard of a randomly chosen individual from an area with higher mortality to the hazard of a randomly chosen individual from an area with lower mortality (Austin et al., 2017; Bengtsson & Dribe, 2010). The Stata ‘mestreg’ command was used for the estimation. We estimated 95% confidence intervals with a non-parametric bootstrap procedure accounting for clustering at municipality level with 250 iterations for all estimates mentioned in the text. We do not provide confidence intervals for all estimates as bootstrap iterations are computationally demanding with the large nested data sets we use. As our analysis is based on total population data, we trust our main results and conclusions to be robust. All analyses were carried out separately for men and women.

First, we calculated municipal-level mortality rates and descriptive statistics for age and socioeconomic characteristics for the first and last period of the follow-up. We then estimated empty models (model 0) for the first and the last period, and obtained predictions of municipality-specific effects (empirical Bayes means of random effects). These estimates are presented in caterpillar plots as hazard ratios (HR) with the average hazard over municipalities as a reference to visually examine the overall change in mortality variation between municipalities before adjusting for any individual-level variables. Second, we present MHRs for empty (model 0), and age-adjusted (model 1) models, and a model further adjusted for socioeconomic characteristics (model 2) for each of the ten periods to investigate the extent to which changes in area variation were explained by changes in municipality age and socioeconomic structures. Thereafter, to examine whether trends varied by age, we estimated MHRs separately for mortality at ages 30–49, 50–69 and 70 and over. Age was adjusted in five-year groups within these categories. We repeated these models for internal and external causes, and finally included both age and all individual socioeconomic characteristics simultaneously (model 2). In the cause specific analysis persons dying of another cause were censored at their time of death. We present MHRs by cause of death adjusted for age (model 1) and for all individual-level variables (model 2) for those aged 30–49 and 50–69 years since, as we show below, variation is highest among these categories. MHRs by cause of death from models 1 and 2 for the age category 70 and over are presented in the Appendix (Table A1).

## 3 Results

**Fig. A1:**
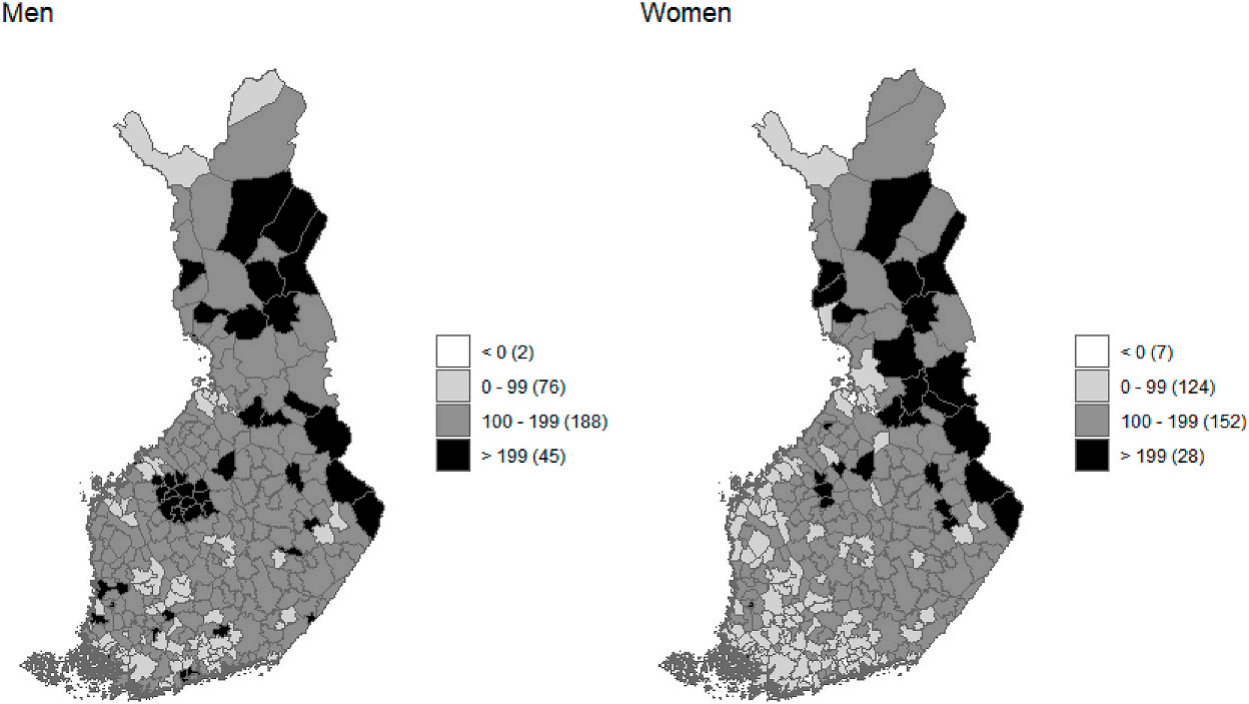
Percentage change between 1972-1975 and 2016–2018 in the share of population 70 years and over, by municipality and sex.

Descriptive statistics (Table 1) show considerable variation in the demographic and socioeconomic structure of Finnish municipalities. There are also significant changes in these underlying determinants of mortality over time. While the entire country is aging, since 1972–1975 the mean age of municipality populations has increased by more than 6 years for both men and women; the dispersion of mean ages across municipalities has also increased. Figure A1 in the Appendix shows that the aging of the population structure has been particularly pronounced in municipalities in the eastern and northern parts of the country. In contrast, the proportion of population with only basic education as well as the share of manual workers have substantially declined over time. For education, there is evidence of increased variation between municipalities, but not for the proportion of manual workers, and those with low income. Over the study period, crude mortality has decreased within each age category, whereas the proportion of deaths attributable to external causes has increased.

**Table 1:** Deaths, person-years, death rates and mean rates by age category across municipalities and municipality means and standard deviations of age and socioeconomic characteristics in 1972–1975 and 2016–2018, men and women aged 30 years and over at baseline.

	1972–1975		2016–2018	
	Men	Women	Men	Women
*Mortality at ages 30 years and over*				
Deaths (% external)	86,263 (9.9%)	75,814 (4.1%)	79,420 (11.8%)	80,411 (5.1%)
Person-years	3,879,123	4,746,334	5,102,822	5,483,753
Death rate (per 1000)	22.2	16.0	15.6	14.7
Municipality mean (sd)	24.0 (4.1)	17.0 (3.8)	19.2 (5.2)	17.9 (5.1)
*Mortality at 30–49 years*				
Deaths (% external)	13,061 (32.7%)	4727 (17.9%)	3758 (50.7%)	1655 (27.6%)
Person-years	2,204,655	2,283,972	2,100,369	2,005,598
Death rate (per 1000)	5.9	2.1	1.8	0.8
Municipality mean (sd)	6.1 (2.1)	2.1 (1.1)	2.2 (2.4)	0.9 (1.3)
*Mortality at 50–69 years*				
Deaths (% external)	44,511 (7.5%)	26,960 (4.3%)	23,492 (19.6%)	12,176 (12.7%)
Person-years	1,422,565	1,954,507	2,171,630	2,263,803
Death rate (per 1000)	31.3	13.8	10.8	5.4
Municipality mean (sd)	31.0 (6.1)	13.6 (3.0)	11.3 (3.4)	5.5 (1.9)
*Mortality at 70 years and over*				
Deaths (% external)	28,691 (3.2%)	44,127 (2.5%)	52,170 (5.5%)	66,580 (3.1%)
Person-years	251,903	507,855	830,823	1,214,352
Death rate (per 1000)	113.9	86.9	62.8	54.8
Municipality mean (sd)	115.2 (19.1)	89.4 (16.5)	66.2 (12.0)	58.4 (10.4)
*Age and socioeconomic characteristics, municipality mean (sd)*				
Age	50.3 (1.8)	52.7 (2.1)	57.0 (2.5)	59.2 (2.8)
Proportion with basic education or less	84.5 (6.9)	85.7 (4.3)	33.7 (7.1)	31.0 (6.1)
Proportion of manual workers	30.5 (10.7)	18.9 (8.6)	19.7 (3.8)	9.0 (1.9)
Proportion in lowest income quintile	26.8 (8.9)	24.8 (5.8)	23.7 (7.1)	22.6 (5.7)

**Fig. 1:**
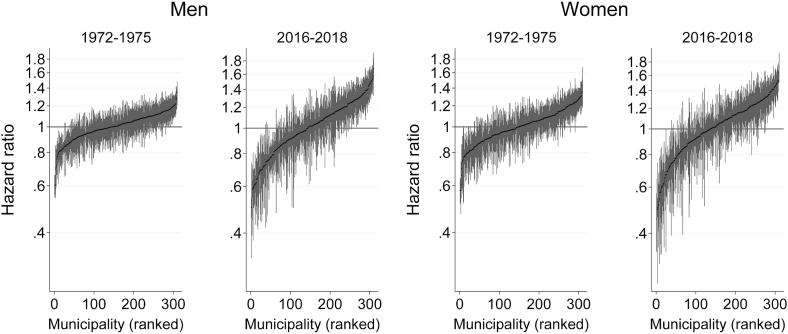
Differentials in mortality across Finnish municipalities in 1972–1975 and 2016–2018 among men and women aged 30 and over, represented by municipality-specific HR and 95% confidence intervals with the reference being the average hazard of all municipalities. Notes: HRs are predicted from empty models (model 0) and y-axis is on log scale. Width of confidence intervals is inversely related to municipality size.

Fig. 1 shows predicted municipality-specific hazard ratios (HR) derived from empty models for 1972–1975 and 2016–2018. The HRs are plotted in ascending order along with 95% confidence intervals and they represent percentage deviations of each municipality relative to the predicted average hazard represented by the horizontal line at one. The overall variation in mortality across municipalities increased over time. For example, the 10th and 90th percentile values of municipality HRs (the point estimates of municipality ranks 32 and 280) in 1972–1975 were 0.86 and 1.13 among men and 0.83 and 1.19 among women, whereas in 2016–2018 the corresponding values were 0.71 and 1.33 for men and 0.70 and 1.32 for women.

**Fig. 2:**
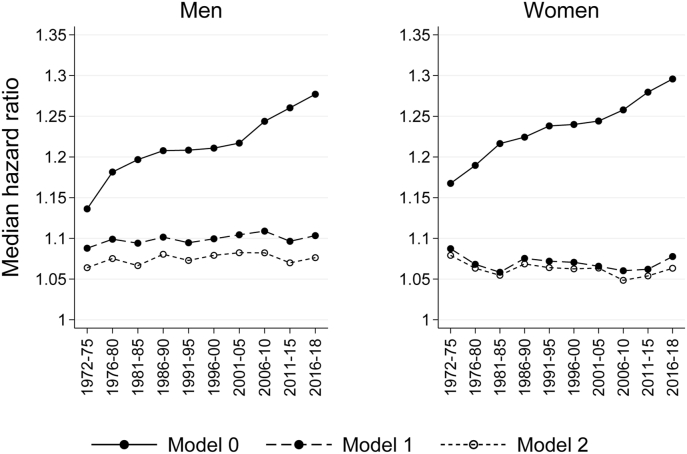
Median hazard ratio (MHR) for 10 periods between 1972-1975 and 2016–2018 from empty (model 0) and age-adjusted (model 1) models, and a model adjusted for age and socioeconomic characteristics (model 2), by sex, population aged 30+.

While the hazard ratios of Fig. 1 show how the hazard in each municipality varied from the average hazard, in Fig. 2 the median hazard ratios (MHR) capture this variability and summarize it into one statistic, thereby permitting the observation of changes in variability over time. The MHRs estimated separately for each of the 10 periods between 1972–1975 and 2016–2018 show that the increase in mortality differentials between municipalities was in large part due to increasing differences in municipality age structures. Before adjusting for age (model 0), the MHR increased from 1.14 (95% CI 1.12–1.15) in 1972–1975 to 1.28 (CI 1.26–1.30) in 2016–2018 among men and from 1.17 (CI 1.15–1.18) to 1.30 (CI 1.27–1.32) among women, the increase being particularly pronounced from around 2000 onwards. For men, this increase implies that when comparing two random individuals from different municipalities, the one living in the municipality with higher mortality had, on average, a 14% higher hazard of dying in the early 1970s, whereas in the late 2010s this hazard had doubled to 28%. However, adjustment for age (model 1) made the upward trend disappear and MHRs fluctuated moderately at lower levels, between the extremes of 1.09 (CI 1.08–1.10) in 1972–75 and 1.11 (CI 1.10–1.12) in 2006–10 for men, and 1.06 (CI 1.05–1.07) in 1981–85 and 1.09 (CI 1.07–1.10) in 1972–75 for women. After adjusting for individual socioeconomic characteristics (model 2) the MHR values were further decreased in a similar manner throughout the entire period, but the change was relatively small, particularly among women.

**Fig. 3:**
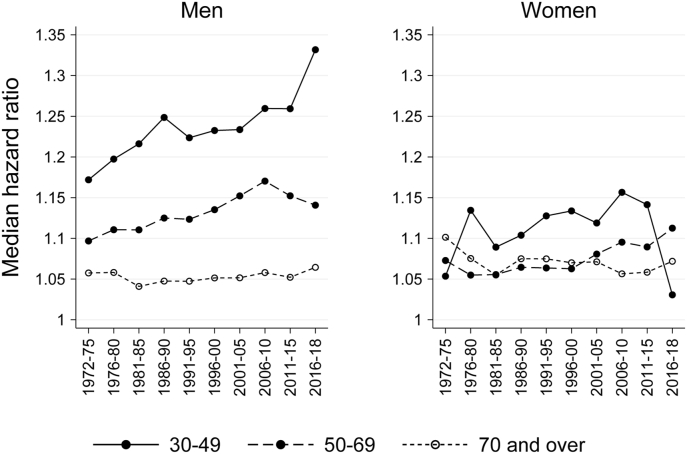
Median hazard ratio (MHR) from age-adjusted models (model 1) for ten periods between 1972-1975 and 2016–2018, by age category and sex.

Although nearly no changes in age-adjusted mortality differentials between municipalities were observed for men and women aged 30 and over, models fitted separately for age categories (Fig. 3) showed that differentials were largest and increased most among younger men ages 30–49: MHR rose from 1.17 (CI 1.14–1.19) in 1972–75 to 1.33 (CI 1.22–1.44) in 2016–18. For men at ages 50–69, MHR also increased until the second half of the 2000s, from 1.10 (CI 1.09–1.12) in 1972–75 to 1.17 (CI 1.15–1.19) in 2006–10, but among men aged 70 years or more there were little to no changes in MHR during the study period and, overall, municipality variation was low among older men compared to younger age categories. The increase observed in younger age categories was not reflected in the overall trend shown in Fig. 2 as the high levels of mortality at older ages dominate the results for all ages. For women, patterns by age category and their trends were less clear, although there was some indication of an increase since 1995 for those aged 50–69 (from 1.06 (CI 1.04–1.09) in 1996–2000 to 1.11 (CI 1.07–1.15) in 2016–18).

Table 2 shows the results from models adjusted for age and socioeconomic characteristics for ages 30–49 and 50–69 for all-cause, internal and external mortality (results for ages 70 and over shown in the Appendix Table A1). For internal causes, differentials between municipalities remained relatively stable over time for both age categories and sexes. For external causes, variation was larger than for internal causes, and increased over time, particularly among men at ages 30–49, from 1.23 (CI 1.16–1.29) in 1972–75 to 1.30 (CI 1.21–1.39) in 2011–15 and 1.29 (CI 1.09–1.50) in 2016–18.

**Table 2:** Median hazard ratios (MHR) for 10 periods between 1972–1975 and 2016–2018 for all-cause, internal and external mortality, men and women aged 30–49 and 50–69 at baseline.

	Model 1			Model 2		
	All-cause	Internal	External	All-cause	Internal	External
*Men*						
*30-49 years*						
1972–1975	1.17	1.16	1.23	1.10	1.09	1.21
1976–1980	1.20	1.17	1.21	1.14	1.08	1.20
1981–1985	1.22	1.17	1.23	1.11	1.07	1.16
1986–1990	1.25	1.18	1.28	1.17	1.13	1.21
1991–1995	1.22	1.16	1.28	1.17	1.12	1.25
1996–2000	1.23	1.16	1.31	1.13	1.10	1.18
2001–2005	1.23	1.11	1.31	1.10	1.06	1.15
2006–2010	1.26	1.15	1.33	1.08	1.06	1.12
2011–2015	1.26	1.22	1.30	1.06	1.06	1.11
2016–2018	1.33	1.20	1.29	1.14	1.10	1.13
*50-69 years*						
1972–1975	1.10	1.10	1.13	1.07	1.07	1.15
1976–1980	1.11	1.11	1.16	1.08	1.07	1.18
1981–1985	1.11	1.10	1.20	1.08	1.06	1.22
1986–1990	1.12	1.12	1.18	1.10	1.08	1.20
1991–1995	1.12	1.12	1.20	1.09	1.09	1.21
1996–2000	1.14	1.12	1.22	1.10	1.09	1.20
2001–2005	1.15	1.13	1.26	1.10	1.09	1.19
2006–2010	1.17	1.15	1.25	1.11	1.09	1.19
2011–2015	1.15	1.14	1.21	1.09	1.07	1.17
2016–2018	1.14	1.11	1.23	1.07	1.06	1.12
*Women*						
*30-49 years*						
1972–1975	1.06	1.01	1.22	1.10	1.09	1.22
1976–1980	1.13	1.10	1.50	1.15	1.11	1.52
1981–1985	1.09	1.07	1.23	1.12	1.09	1.29
1986–1990	1.10	1.07	1.24	1.14	1.12	1.27
1991–1995	1.13	1.11	1.26	1.13	1.11	1.29
1996–2000	1.13	1.08	1.31	1.13	1.08	1.29
2001–2005	1.12	1.12	1.22	1.09	1.09	1.19
2006–2010	1.16	1.11	1.29	1.12	1.08	1.20
2011–2015	1.14	1.09	1.30	1.04	1.00	1.15
2016–2018	1.03	1.12	1.17	1.00	1.12	1.07
*50-69 years*						
1972–1975	1.07	1.07	1.13	1.06	1.06	1.15
1976–1980	1.05	1.06	1.17	1.07	1.06	1.20
1981–1985	1.06	1.05	1.18	1.07	1.06	1.25
1986–1990	1.06	1.06	1.24	1.07	1.07	1.29
1991–1995	1.06	1.07	1.21	1.08	1.08	1.26
1996–2000	1.06	1.06	1.20	1.08	1.08	1.24
2001–2005	1.08	1.07	1.21	1.10	1.09	1.24
2006–2010	1.10	1.08	1.26	1.10	1.09	1.27
2011–2015	1.09	1.08	1.16	1.09	1.08	1.19
2016–2018	1.11	1.11	1.17	1.07	1.08	1.12

Model 1: age. Model 2: age, education, occupational social class, and income.

After adjusting for individual socioeconomic characteristics, the municipal-level differences were substantially attenuated among men, for all-cause mortality and for both cause of death groups but especially for external causes in the 2000s. Among women, this adjustment did not attenuate the estimates as in the case of men, but in the last periods studied MHR also slightly decreased when socioeconomic characteristics were considered.

## 4 Discussion

Using individual-level data on the total Finnish population spanning nearly 50 years, we explored long-term trends in municipal-level mortality variation and the contribution of individual compositional factors. The results show a substantial increase in variation in all-cause mortality over time. However, adjustment for age revealed that virtually all the increase was accounted for by increasing differentiation in municipal age structures and, in effect, municipal-level variation in age-adjusted mortality has remained relatively stable since the 1970s. Similar results of persisting differentials between larger areas of the country have been documented in an earlier study (Wilson et al., 2020).

Despite the overall stability, age-specific results showed that at younger ages the differentials have grown wider. The greatest increases were found for men aged 30–49. At ages 70 years and older, variation remained stable throughout the period for both men and women. Adjustment for socioeconomic characteristics further reduced the mortality differentials between municipalities in all periods studied, but more so for men than for women. The attenuation was especially pronounced among men aged 30–49 from around mid-1990s onwards, suggesting that the importance of the socioeconomic population composition of municipalities in explaining within-country area variation also increased during the study period, and particularly for men.

Overall, our findings draw attention to the increased contribution of compositional factors – especially in terms of age, but also socioeconomic characteristics – in area-level mortality variation. We interpret these trends in light of the underlying macro-level developments of past decades. In Finland, recessionary periods, industrial restructuring and increasing unemployment have hit some areas harder than others and led to increasing regional differentiation with important implications for population structures at subnational levels (Grunfelder et al., 2020). Remote rural municipalities and smaller towns in particular have been losing young and working-age people moving to urban centres with better education and employment prospects. The Finnish population is among the most mobile in Europe in terms of internal migration (Grunfelder et al., 2020). Finland is also one of the fastest ageing countries (United Nations, Department of Economic and Social Affairs, Population Division, 2019), and the effects of an ageing population structure are felt especially severely in municipalities with high out-migration (Grunfelder et al., 2020; Stjernberg and Penje, 2019), developments also observed in other countries. These demographic processes are echoed in our findings – overall mortality variation between areas has increased as some municipalities are ageing faster than others due to youth out-migration – and provide support for the interpretation that the effects of restructuring have increased the importance of individual compositional factors in explaining area-level mortality variation.

Highlighting the contribution of compositional changes is important not least because composition and context operate together (Macintyre & Ellaway, 2003). Local context determines the opportunity structure available to residents (Macintyre et al., 2002), and the impact of economic restructuring may increase local disadvantage in municipalities with high out-migration (Strohmeier & Silvia, 2004). Furthermore, the consequences may be felt most acutely among certain population groups (Franklin, 2021). Younger residents in particular are exposed to limited job markets, inadequate educational opportunities and weakened social capital structure. Such effects may be reflected in adoption of risky behaviour, as our results of increased variation in external mortality suggest. Further research to identify specific area characteristics that may have contributed to these trends on the one hand, and assessment of the spatial patterning of the variation on the other, are needed to help clarify the processes at play.

**Fig. A2:**
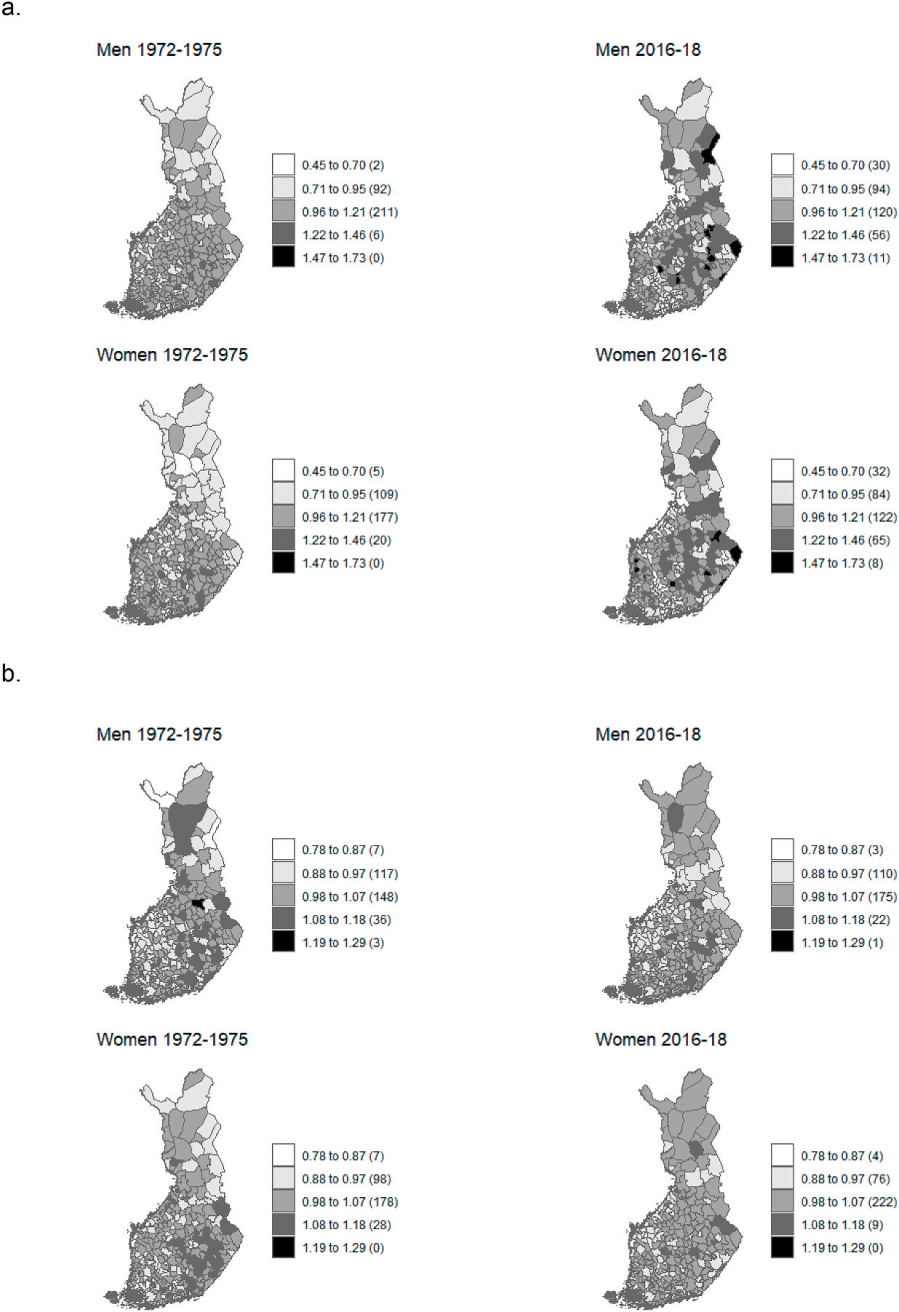
Municipality specific hazard-ratios in 1972–1975 and 2016–2018 among men and women aged 30 and over. Maps in the upper panel (a) display hazard ratios predicted from unadjusted models (model 0) and maps in the lower panel (b) are from models adjusted for age and socioeconomic characteristics (model 2).

The impact of migration on within-country mortality differentials has been acknowledged in many previous studies. Evidence on the direction of the impact is, however, mixed. Studies from Britain support the idea that migration accounts for a significant part of the increase in mortality differentials between areas (Brimblecombe et al., 1999; Norman et al., 2005), whereas evidence from the US in contrast suggests that differentials have been attenuated by migration (Ezzati et al., 2008). The impact of migration on shaping area mortality differentials is based on its selectivity. This selectivity has been argued to operate mainly through sociodemographic selection (Jongeneel-Grimen et al., 2011, Vaalavuo & Sihvola, 2021) – as the propensity to move is higher among those who are younger – but also directly through health as according to many studies those who move are healthier than those who stay (Luy & Gazelli, 2007). Previous research on regional mortality variation in Finland has consistently shown that mortality in the northern and eastern parts of the country is higher compared to regions located in southern and western Finland (Wilson et al., 2020). Our results are in line with these findings as is shown in the maps of Figure A2 in the Appendix. These maps show the spatial patterning of the municipality specific hazard ratios displayed in Fig. 1 classified into five intervals of equal length across periods and sexes. All in all, municipalities with highest hazard ratio values are mostly located in the eastern and – to a lesser extent – northern parts of the country. The maps displaying unadjusted figures clearly show the increase in the number of municipalities at the extremes of the distribution. In these maps also the main urban centres and their surrounding areas appear more clearly as areas of low mortality in the latter period, which is explained by the migration of young people to these areas.

While many previous studies provide evidence of increasing area differentials in mortality (Fenelon, 2013; Leyland, 2004; Montero-Granados et al., 2007; Mustard et al., 1999; Pearce & Dorling, 2006; Wang et al., 2013), studies reporting stagnation or declines in differentials also exist (Gächter & Theurl, 2011; Janssen et al., 2016; Skaftun et al., 2018). It is likely that the diversity arises from differences in population structures and social contexts but probably also from heterogeneity in methodological choices. Our approach draws attention to the role of changes in population composition of areas, an aspect not addressed in previous studies. We present unadjusted figures as they describe the actual challenges health care systems are facing locally and also because if we only analysed age-standardized measures important mechanisms behind regional mortality disparities and their trends would remain unacknowledged and the regional policy impact of ageing populations unrecognized. This impact is likely to be particularly challenging for the aging municipalities in terms of financing and organisation of hospital and elderly long-term care (Murphy & Martikainen, 2013).

Although in this respect our approach differs from most previous studies and making direct comparisons with existing research is therefore difficult, some interesting parallels to previous findings can be identified, in particular with regards to younger age categories. A study looking at state-level differentials in mortality in Germany between 1991 and 2015 (van Raalte et al., 2020) also found increasing variation among working-age men. Although this study did not consider causes of death, the role of health behaviour as a key driver of changes in within-country mortality variation has been observed in previous studies (Vierboom et al., 2019; Muszyńska et al., 2014). These studies have pointed out that differences and changes in smoking behaviour over time may largely explain mortality differentials between areas and their trends.

We found higher area differentials for external causes of death, and although for internal causes variation remained relatively stable over time, for external causes it grew wider. The growth was particularly evident, again, for men aged 30–49, and this was largely responsible for the increase in variation observed for all-cause male mortality in this age category. Since among women deaths due to external causes were much rarer, the modestly increasing variation in external mortality did not affect all-cause female variation, which explains the lack of similarity with the trends and age patterns observed among men. Based on previous evidence from Finland, alcohol-related mortality is known to significantly contribute to differences in all-cause mortality between different areas of the country (Mäkelä et al., 2001). Although alcohol-related deaths have a determinant role in the mortality of the working-age, particularly male, population and the adverse health effects of alcohol consumption, including mortality, have been reported to be increasing (Mäkelä, 2003) we are not aware of studies examining changes in regional variation in alcohol-related mortality over time. Our results suggest that this variation may have increased.

**Fig. A3:**
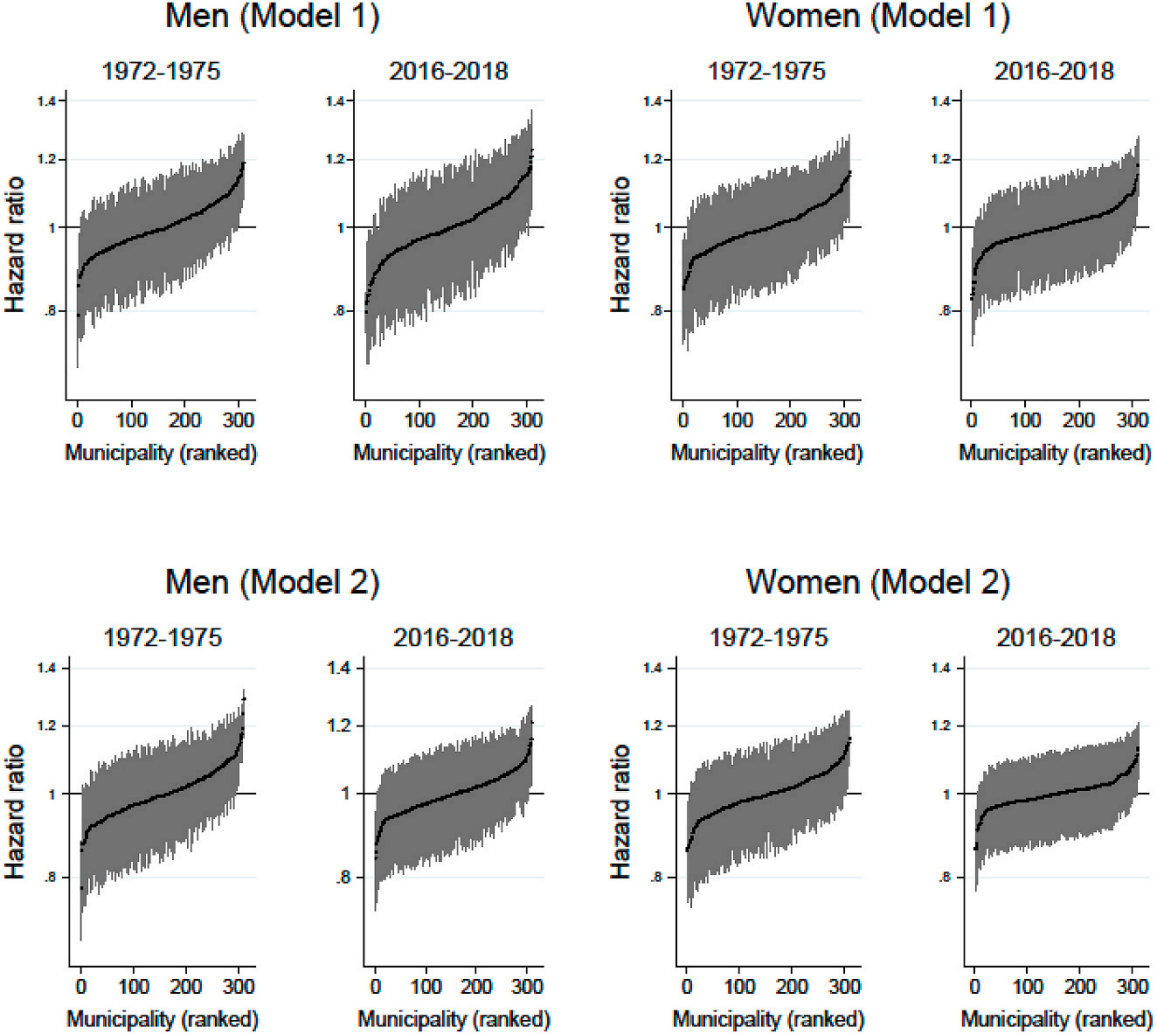
Differentials in mortality across Finnish municipalities in 1972–1975 and 2016–2018 among men and women aged 30 and over, represented by municipality-specific HR and 95% confidence intervals predicted from models adjusted for age (model 1) and for age and socioeconomic characteristics (model 2).

Key strengths of this study include the total individual-level population data, detailed municipal-level data, and the long study period covering nearly five decades. The data is based on high-quality registers that have minimal attrition and are not subject to underreporting or recall bias. These data characteristics permitted us to adopt a multilevel design and to adjust for individual-level covariates and assess their role in long-term change. We used the median hazard ratio (MHR) as our summary measure to examine changes in the magnitude of municipal level mortality variation. The advantage of using this relatively new measure is that it provides a concise measure to assess whether mortality variation across municipalities has changed over time while accounting for individual-level characteristics. A possible disadvantage is that an increasing number of outliers (e.g. single municipalities with high excess mortality) may drive changes in the overall estimate. Caterpillar plots of municipality hazard ratios predicted from models 1 and 2 however rule out this possibility as no major outliers appear between periods (see Figure A3 in the Appendix).

The choice of area unit is an issue that deserves some thought. We studied variation at the municipality level as we believe it is a valid unit of analysis when it comes to mortality since it holds administrative authority in the health sector. Additionally, it is the smallest unit available in our data, and it has been suggested that area effects vary inversely according to the size of the area – smaller areas are less heterogeneous, which increases variation between areas (Tarkiainen et al., 2010). As a sensitivity analysis, we tested whether the exclusion of the largest municipalities (pop>100,000) significantly changed the results. However, findings were similar, and we thus show results incorporating all municipalities. Furthermore, since previous results on mortality differentials in the Finnish capital region suggest that the choice of area unit does not significantly affect the estimation of area effects (Tarkiainen et al., 2010), we believe our main results are robust to the area level chosen. Nevertheless, further longitudinal studies using different area definitions are needed.

In conclusion, our results suggest that, conditional on age, overall municipal-level variation in all-cause mortality has remained relatively stable in Finland over a period of five decades. A possible explanation relates to the Finnish welfare system aiming at securing equal health and social care regardless of place of residence and policies to mitigate the consequences of economic restructuring. While these policies seem to have been successful among the elderly as increased age structure differences between municipalities and diverging health care needs and expenditures have not coincided with increased area variation in mortality in the oldest age group, the findings regarding younger men is a source of concern. Similarly, our results provided no evidence of decreasing variation, which would be ideal from an equity perspective. Moreover, there is not much reason to expect mortality differentials between areas to decrease since spatial differentiation is likely to continue as the population continues concentrating in a few growth centres. This will have important implications for future planning since policy measures and interventions need to consider how to meet the ever more divergent service needs of municipalities.

## Author statement

**Ulla Suulamo:** Conceptualization, Methodology, Software, Formal analysis, Investigation, Writing - Original Draft, Writing - Review & Editing, Visualization **Lasse Tarkiainen:** Conceptualization, Methodology, Software, Writing - Review & Editing **Hanna Remes:** Conceptualization, Methodology, Writing - Review & Editing **Pekka Martikainen:** Conceptualization, Methodology, Writing - Review & Editing.

## Funding

This work was supported by the 10.13039/501100002341Academy of Finland [grant numbers 1294861, 1308247].

## Ethical statement

The study has been approved by Statistics Finland Board of Statistical Ethics. As the data were collected for routine administrative registration purposes informed consent from participants was not required.

The manuscript is based on original research of the authors. The manuscript is not currently being considered for publication elsewhere. We confirm that the order of authors listed in the manuscript has been approved by all of us and that all authors have read and approved the final version of the manuscript.

There is no financial or other conflict of interest that might have biased our work.

## Declarations of competing interest

None.

## Figures and Tables

**Table A1:** Median hazard ratios (MHR) for 10 periods between 1972–1975 and 2016–2018 for all-cause, internal and external mortality, men and women aged 70 and over at baseline.

	Model 1			Model 2		
	All-cause	Internal	External	All-cause	Internal	External
*Men*						
*70 years and over*						
1972–1975	1.06	1.06	1.26	1.06	1.06	1.26
1976–1980	1.06	1.06	1.12	1.06	1.06	1.14
1981–1985	1.04	1.04	1.10	1.05	1.04	1.13
1986–1990	1.05	1.04	1.14	1.05	1.05	1.15
1991–1995	1.05	1.05	1.14	1.04	1.04	1.15
1996–2000	1.05	1.05	1.16	1.05	1.05	1.18
2001–2005	1.05	1.05	1.14	1.07	1.06	1.17
2006–2010	1.06	1.06	1.13	1.06	1.06	1.15
2011–2015	1.05	1.05	1.15	1.06	1.06	1.18
2016–2018	1.06	1.06	1.09	1.06	1.05	∗
*Women*						
*70 years and over*						
1972–1975	1.10	1.10	1.08	1.10	1.10	1.07
1976–1980	1.08	1.08	1.27	1.07	1.08	1.27
1981–1985	1.06	1.06	1.13	1.06	1.06	1.14
1986–1990	1.08	1.07	1.20	1.07	1.07	1.19
1991–1995	1.07	1.08	1.22	1.07	1.07	1.22
1996–2000	1.07	1.07	1.30	1.07	1.06	1.30
2001–2005	1.07	1.07	1.19	1.07	1.07	1.19
2006–2010	1.06	1.06	1.16	1.05	1.05	∗
2011–2015	1.06	1.06	1.19	1.05	1.06	∗
2016–2018	1.07	1.07	1.12	1.06	1.07	∗

Model 1: age. Model 2: age, education, occupational social class, and income.

∗ Models failed to converge given low number of deaths.
